# Comparison of Radiographic and Patient-Reported Outcomes After Surgery in Adolescent Idiopathic Scoliosis Between Robotics and Navigation: An Analysis Using Propensity Score Matching

**DOI:** 10.7759/cureus.49061

**Published:** 2023-11-19

**Authors:** Tsutomu Akazawa, Yoshiaki Torii, Jun Ueno, Masahiro Iinuma, Atsuhiro Yoshida, Ken Tomochika, Takahiro Hideshima, Seiji Ohtori, Hisateru Niki

**Affiliations:** 1 Department of Orthopaedic Surgery, St. Marianna University School of Medicine, Kawasaki, JPN; 2 Spine Center, St. Marianna University Hospital, Kawasaki, JPN; 3 Department of Orthopedic Surgery, Graduate School of Medicine, Chiba University, Chiba, JPN

**Keywords:** robotic-assisted pedicle screw placement, navigation, robotics, pedicle screw, adolescent idiopathic scoliosis

## Abstract

Purpose

This study aimed to compare the radiographic and patient-reported outcomes after surgery in adolescent idiopathic scoliosis (AIS) between robotics and navigation using propensity score matching.

Methods

This retrospective study involved 50 patients undergoing posterior spinal fusion for AIS between October 2016 and August 2022, utilizing navigation or robotic systems, analyzing them using propensity score matching. The evaluations included assessments using X-ray, Scoliosis Research Society 22-Item (SRS-22) Questionnaire, and CT, considering variables such as age, gender, BMI, and Lenke type.

Results

Post matching, 13 cases each from robotics and navigation groups were compared. No significant differences were found in the demographic variables, preoperative X-ray parameters, and preoperative SRS-22 scores between the two groups. The robotics group demonstrated a higher perfect accuracy rate (94.0% vs. 84.7%, p=0.005) and a lower deviation rate in pedicle screw placements (1.6% vs. 4.1%, p=0.223). At one year postoperatively, there were no significant differences in the X-ray parameters between both groups. Likewise, no significant differences were found in each domain of SRS-22, but function, self-image, mental health, and satisfaction scores were numerically higher in the robotics group.

Conclusion

The application of a spinal robotic system in AIS surgery presented enhanced screw accuracy and lower deviation rates, compared to navigation, with no significant differences observed in the X-ray parameters and each domain of SRS-22 at one year postoperatively. This suggests that, to improve patient quality of life (QOL), it is essential for robotic-assisted spine surgery to focus not only on screw accuracy but also on the development of novel robotic-assisted techniques.

## Introduction

Meta-analysis has reported that the robotic-assisted pedicle screw placement has higher accuracy (odds ratio 1.68) and fewer complications (odds ratio 0.31) compared to the conventional freehand technique [1]. Patients undergoing one-three-level robotic-assisted lumbar posterior fusion for degenerative diseases have been reported to have no increased complication rate at 90 days and significantly shorter hospital stay compared to patients not receiving robot assistance [2]. The application of robotic-assisted spine surgery potentially results in reduced intraoperative blood loss, reduced reoperations, decreased infection rate, reduced hospital stay, and shortened surgery time [3,4].

Robotics have been reported to be particularly useful tools for enhancing screw placement accuracy for surgeons with less experience in spinal deformity surgery [5]. Regarding the accuracy of robotics in adolescent idiopathic scoliosis (AIS), high accuracy ranging from 92.8% to 98.7% has been reported [6-9]. Despite the use of robotics improving the accuracy of screw placement in our previous study [9], it has not been reported how it affects the radiographic and patient-reported outcomes.

The objective of this study is to compare the radiographic and patient-reported outcomes after surgery in AIS between robotics and navigation. We investigated the X-ray parameters and the results related to health-related quality of life (QoL) and used propensity score matching to compare robotics with navigation.

## Materials and methods

Subjects

The Institutional Review Board of our institution approved this retrospective study (approval no. 6138). The subjects were 50 patients who underwent posterior spinal fusion for AIS from October 2016 to August 2022. The inclusion criteria were as follows: 1) AIS, 2) age at the time of surgery between 10 and 25 years, and 3) posterior spinal fusion using pedicle screws. The exclusion criteria included 1) congenital scoliosis, 2) neuromuscular scoliosis, 3) syndromic scoliosis, and 4) cases with anterior spinal fusion. There were 42 females and eight males, with an average age at the time of surgery of 15.8 years. According to the Lenke classification, there were type 1 (25 cases), type 2 (eight cases), type 3 (four cases), type 5 (11 cases), and type 6 (two cases). These were 50 consecutive cases, divided into two groups according to the time period. Up until April 2021, preoperative CT-based navigation (Stealth Station S7, Medtronic Inc., Dublin, Ireland) was used in consecutive 32 cases (navigation group), and subsequently, a spinal robotic system (Mazor X Stealth Edition, Medtronic Inc., Dublin, Ireland) was used in consecutive 18 cases from May 2021 to August 2022 (tobotics group).

Surgical workflow

Preoperative CT images were used to perform screw placement planning in each dedicated application. The same surgical team performed the surgeries in both groups.The surgery is conducted in the prone position, and the fixation range is approached through a midline skin incision.

Navigation Group

A reference frame was installed on the most caudal registered vertebra, and multilevel registration was conducted for three to four vertebrae [10]. In one registration, pedicle screws were placed for up to five vertebrae. Using navigation, probing, tapping, and screw placement were performed.

Robotics Group

**Figure 1:**
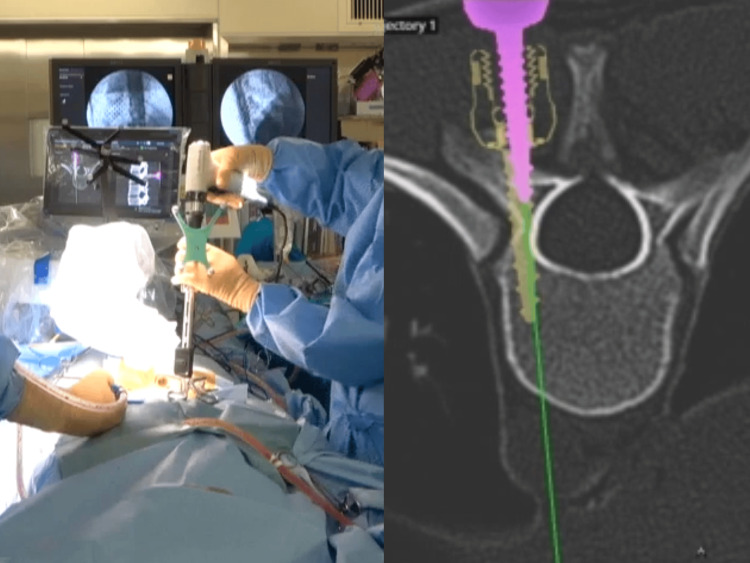
Pedicle screws were placed under the robotic arm guide and could be viewed on the screen.

The robotic arm unit was connected with a clamp placed on the spinous process of the most caudal vertebra using bone mount, and the robot reference frame was utilized. Preoperative CT images were matched with intraoperative C-arm images using CT-to-fluoro registration. In one registration, screws were placed for up to seven vertebrae. Drilling, tapping, and screw placement were performed under the robotic arm guide and could be viewed on the screen (Figure 1). In both groups, after screw placement, correction was performed using cantilever technique with two rods and then fixed. Closure was performed with standard manners.

Evaluations

Radiographic Outcome

Plain radiographs were taken in an upright position, including frontal and lateral views of the entire spine. Evaluations were made preoperatively and one year postoperatively. Preoperatively, entire spine side-bending radiographs were taken in a supine position. X-ray parameters were measured as follows: In the frontal view, Cobb angles of the proximal thoracic curve (PT), main thoracic curve (MT), and lumbar curve (L) were measured. The curve with the largest Cobb angle was considered the major curve, and its curve angle was termed the major Cobb angle. In the lateral view, thoracic kyphosis (TK: T5-T12) was measured. The ratio of the major bending Cobb angle to the major Cobb angle in standing was considered the flexibility index.

Patient-Reported Outcome

The Scoliosis Research Society-22 Questionnaire (SRS-22) was evaluated preoperatively and one year postoperatively [11,12].

Screw Accuracy

Pedicle screw placements were evaluated in the one-week postoperative CT images using the Gertzbein-Robbins grade: Grade A (no breach of the cortical layer of the pedicle), Grade B (breaches less than 2 mm), Grade C (breaches of 2 mm or more but less than 4 mm), Grade D (breaches of 4 mm or more but less than 6 mm), and Grade E (breaches of 6 mm or more) [13]. Pedicle screw placement was assessed on the computer screen by one author (J.U.) who was blinded to the clinical symptoms. The perfect accuracy rate was defined as the proportion of Grade A. Deviations of less than 2 mm were considered within the acceptable range, and the deviation rate was considered the proportion of Grades C, D, and E.

Propensity score matching analysis

The use of robotics was treated as the dependent variable in the propensity score matching, with covariates being age, gender, body mass index, Lenke type, major Cobb angle, major bending Cobb angle, and flexibility index.

Statistical analysis

Continuous variables are presented as mean ± standard deviation. The differences between the robotics and navigation groups were analyzed using unpaired t-tests, χ2 tests, or Fisher's exact test, as appropriate. A p-value less than 0.05 was considered to indicate a statistically significant difference.

## Results

As a result of the matching, 13 cases were extracted for the robotics group and 13 cases for the navigation group. There were no significant differences between the two groups in terms of age, gender, BMI, and Lenke type. There were no significant differences between the two groups in the number of vertebrae fixed, blood loss, and operative time. There were no significant differences in PT, MT, L, TK, Major Cobb angle, major bending Cobb angle, and flexibility index preoperatively. In preoperative SRS-22, there were no significant differences between the two groups in function, pain, self-image, and mental scores (Table 1). There were no cases with neurological deficits in either group.

**Table 1:** Background factors, preoperative X-ray parameters, and SRS-22 of the robotics and navigation groups. Variables are presented as mean ± standard deviation. PT; proximal thoracic curve, MT; main thoracic curve, L; lumbar curve, TK; thoracic kyphosis; SRS-22: Scoliosis Research Society-22 Questionnaire

	Robotics group (n=13)	Navigation group (n=13)	p
Age (years)	16.2 ± 2.5	15.8 ± 1.8	0.657
Gender (% of female）	84.6%	84.6%	1.000
Body mass index	18.5 ± 2.6	18.6 ± 1.5	0.847
Lenke type			0.875
1	7	8	
2	3	3	
3	0	0	
4	0	0	
5	3	2	
6	0	0	
Number of fused vertebrae	9.2 ± 2.4	9.2 ± 2.2	0.931
Intraoperative blood loss (ml)	293.1 ± 167.0	335.2 ± 367.4	0.710
Operative time (min)	295.8 ± 58.1	262.2 ± 44.1	0.110
Preoperative X-ray parameters			
PT	22.0 ± 12.5	26.2 ± 5.2	0.279
MT	44.2 ± 14.3	47.4 ± 8.3	0.497
L	34.6 ± 10.1	30.9 ± 10.4	0.369
TK	20.9 ± 9.9	18.6 ± 9.7	0.554
Major Cobb angle	50.0 ± 7.4	48.8 ± 5.1	0.648
Flexibility index (%)	52.8 ± 17.2	51.9 ± 11.9	0.878
Preoperative SRS-22			
Function	4.8 ± 0.3	4.8 ± 0.3	0.788
Pain	4.4 ± 0.7	4.5 ± 0.6	0.839
Self-image	2.9 ± 0.5	3.0 ± 0.5	0.810
Mental health	4.2 ± 0.5	4.0 ± 0.5	0.409

At one year postoperatively, there were no statistically significant differences between the two groups in PT, MT, L, and TK. However, MT was numerically smaller in the robotics group (robotics group: 17.2±5.0 degrees, navigation group: 19.5±3.4 degrees; p=0.166), and TK was numerically smaller in the robotics group (robotics group: 21.4±6.1 degrees, navigation group: 25.1±4.6 degrees; p=0.095) (Table 2). At one-year postoperative SRS-22, there were no statistically significant differences between the two groups in function, pain, self-image, mental health, and satisfaction scores, but the robotics group had slightly higher tendency SRS-22 scores indicating better outcomes in function, self-image, mental health, and satisfaction (Table 2).

**Table 2:** Comparison in one-year postoperative X-ray parameters and SRS-22. Variables are presented as mean ± standard deviation. PT; proximal thoracic curve, MT; main thoracic curve, L; lumbar curve, TK; thoracic kyphosis; SRS-22: Scoliosis Research Society-22 Questionnaire.

	Robotics group	Navigation group	p
Postoperative X-ray parameters			
PT	15.0 ± 8.6	14.2 ± 4.6	0.778
MT	17.2 ± 5.0	19.5 ± 3.4	0.166
L	12.9 ± 8.2	14.6 ± 4.1	0.512
TK	21.4 ± 6.1	25.1 ± 4.6	0.095
Major Cobb angle	18.4 ± 7.7	19.2 ± 4.1	0.752
Postoperative SRS-22			
Function	4.7 ± 0.3	4.4 ± 0.5	0.146
Pain	4.4 ± 0.4	4.4 ± 0.5	0.927
Self-image	4.0 ± 0.9	3.8 ± 0.5	0.421
Mental health	4.3 ± 0.3	4.2 ± 0.8	0.668
Satisfaction	4.4 ± 0.6	4.2 ± 0.7	0.547

The perfect accuracy rate was significantly higher in the robotics group (robotics group: 94.0%, navigation group: 84.7%; p=0.005). The deviation rate was lower in the robotics group (robotics group: 1.6%, navigation group: 4.1%; p=0.223). In both groups, there were no reoperations due to screw malposition (Table 3).

**Table 3:** Gertzbein–Robbins grade distribution and screw accuracy.

	Robotics group (183 screws)	Navigation group (196 screws)	p
Gertzbein-Robbins grade			
A	172	166	
B	8	22	
C	3	5	
D	0	3	
E	0	0	
Perfect accuracy rate	94.0%	84.7%	0.005
Deviation rate	1.6%	4.1%	0.223

## Discussion

In AIS, the perfect accuracy rate of robotics was significantly superior compared to navigation, and although there was no significant difference in the deviation rate, it was lower in the robotics group. At one year postoperatively, there were no significant differences in X-ray parameters between both groups. Likewise, no significant differences were found in each domain of SRS-22, but the scores were slightly higher in the robotics group. 

Segmental pedicle screw fixation is the most popular procedure in AIS surgery, but it has been reported that pedicle screw placement with the freehand technique is prone to deviation. In comparison between patients with scoliosis and trauma patients, it has been reported that the cortical perforation of pedicle screws in the lumbar spine was statistically more common in patients with scoliosis [14]. Meta-analysis has reported that the deviation rate is higher in the freehand technique compared to the use of CT-based navigation [15]. The deviation rate in scoliosis has been reported to be 1.5% to 11.4% with navigation [10,16-19] and 1.3 to 7.2% with robotics [6-9]. In our study, although there was no significant difference in the deviation rate, the perfect accuracy rate of robotics was significantly superior to navigation.

Computer-aided technologies, such as robotics and navigation, contribute to the safety of pedicle screw placement. Currently, returning to the operating room due to screw malposition is indeed deemed unacceptable. With the use of intraoperative CT navigation, the need to return to the operating room due to screw deviation has never been less [20]. However, improvements in screw placement accuracy do not immediately translate into radiological outcomes or improvements in patient QoL. In our study, there were also no differences in radiological outcomes or patient QoL between robotics and navigation. Minimally invasive spine surgery using intraoperative CT navigation has been reported as an effective and safe alternative to open surgery for AIS patients with Lenke type 5C, showing significantly higher scores in pain and self-image domains using the SRS-22 in the minimally invasive surgery group compared to the open surgery group [21]. The spinal robotic system currently available are specialized in pedicle screw placement. While the screw accuracy has the potential to reduce surgical complications, its effect on improving patient-reported outcomes was limited. Improvements in patient-reported outcomes may be achieved if the robotic system performs other techniques in scoliosis surgery. By not only using robotics but also improving surgical methods by adding minimally invasive techniques, we may contribute to improving patient QoL.

This study has several limitations. It was not a randomized controlled trial. The number of patients, especially in the robotics group, was small. As this is a retrospective study, the number of patients or the number of screws placed in each group was not equal. Although patients were divided into two groups according to the time period, there was a possibility of selection bias. It was also possible that cases with smaller Cobb angles and lower complexity were assigned to specific groups. Therefore, a propensity score matching analysis was used. One year is too early to assess patient-reported outcome measures (PROMs) in a case like AIS. The observation period for this study was only one year, so longer observations are needed in the future. We believe that achieving a perfect accuracy rate not only eliminates the possibility of nerve damage within the deviation tolerance but also means successful screw insertion according to the plan. Achieving planned screw insertion could potentially lead to the development of new insertion methods in the future. Spinal robotic systems are very expensive, and the cost-effectiveness of the improvements in the radiographic and patient-reported outcomes and potential safety needs to be considered.

## Conclusions

In AIS surgery, pedicle screw placement by the spinal robotic system had improved screw accuracy and a lower deviation rate compared to navigation. There were no significant differences in the X-ray parameters and each domain of SRS-22 at one year postoperatively. This suggests that, to improve patient QOL, it is essential for robotic-assisted spine surgery to focus not only on screw accuracy but also on the development of novel robotic-assisted techniques.
